# Culture, eating behavior, and infectious disease control and prevention

**DOI:** 10.1186/s42779-020-00076-y

**Published:** 2020-11-25

**Authors:** Mingzhu Zhou, Na Zhang, Man Zhang, Guansheng Ma

**Affiliations:** 1 Department of Nutrition and Food Hygiene, School of Public Health, Peking University, 38 Xue Yuan Road, Hai Dian District, Beijing, 100191 China; 2 Laboratory of Toxicological Research and Risk Assessment for Food Safety, Peking University, 38 Xue Yuan Road, Hai Dian District, Beijing, 100191 China

**Keywords:** Culture, Eating behavior, Infectious diseases

## Abstract

Humans need to obtain nutrients from foods for survival and health. Culture and belief play important roles in food selection and intake. Throughout human history, dietary factor has been one of the important factors inducing and causing outbreaks of infectious diseases. If unhealthy eating behavior, like eating raw/undercooked food or meat and products from wild animals, are not abandoned, foodborne infectious diseases will remain an important risk factor of outbreaks and epidemics. The misconception of dietary culture is one of the important factors that triggers unhealthy eating behavior. Therefore, it is vital to change people’s conceptions and knowledge about what is healthy to eat, in order to completely eliminate unhealthy eating behavior and prevent the recurrence of foodborne infectious diseases. Meanwhile, many factors such as family, society, region, and religion should be involved in.

## Background

Infectious diseases threaten the health of human beings and bring a serious burden of disease as well [1]. Potential pathogens causing infectious diseases are various, including prion, bacteria, viruses, fungi, rickettsia, spirochete, and parasites [2, 3]. It is estimated that 17 million people die worldwide each year from infectious diseases [4]. Collectively, AIDS, tuberculosis, malaria, hepatitis, and neglected tropical diseases accounted for an estimated 4.3 million deaths in 2016 [5]. Certainly, the emergence and transmission of infections is usually linked with a number of factors [6], such as age [7], gender [8], occupation [9], education level [10], personal behavior [11], climate [12], geographical condition [13], hygienic condition, economic condition [14], massive urbanization, and cultural custom. Since the twenty-first century, a total of seven public health emergencies have been identified by the World Health Organization (WHO) as Public Health Emergency of International Concern (PHEIC), and five of them were associated with eating factors. Thus, it can be seen that eating behavior plays a significant part in the occurrence of infectious diseases. It is meaningful to change people’s conceptions and knowledge about dietary culture and eating behavior.

## Changes in dietary culture and eating behavior

Before human beings learned to use fire, they had spent a long time in the dark. In the primitive life of picking and hunting, everything they ate was raw and grasped directly by hand, including birds and animals, clams and fish, stems, leaves, and fruits. The use of fire opened the door to civilization and gave birth to our human unique culinary skills. Ancient humans ate food cooked by natural fire by accident and found that it was more delicious than raw food, and since then, natural fire was used. Finally, they knew how to create fires through long-term attempts, and learned how to control fire and cook with fire. At the very beginning, people put food directly on the fire. Then, they learned to use a medium to conduct heat in order that the food was evenly heated, such as placing the food on a hot stone, or packing the food with grass or mud or animal skin before using the fire to burn it, or burying the raw materials in the hot ashes or stones to make it cooked. Gradually, cooking utensils emerged during the labor practices, and various ways to preserve and store food were invented.

Backer has mountain, draft relying on water; there are huge differences in dietary culture among different regions around the world. More than 2400 years ago, the Chinese medicine classics the Yellow Emperor’s Internal Classics (Huang Di Nei Jing) proposed grain for raising, fruits for help, meats for benefit, and vegetables for supplement; various foods cannot conflict with each other, so as to replenish vital energy, and grain, meat, and vegetables can provide nutrients, but they cannot be eaten too much, so as not to damage the body. This may be the earliest dietary guideline in the world. The ancient Greek Hippocrates stated that health can only be ensured by proper diet and hygiene and to treat your food like medicine, not your medicine like food. Each nation has its own set of standardized eating etiquette in its long-term practice. Likewise, different religions have also formed their own set of dietary culture. For instance, Taoism advocates vegetarianism and less food, and stopping diet. Buddhism insists that when you get a diet, do not choose it; as long as it can support your body and you can practice the Tao, it is in line with the Buddha. Islamic diet generally follows the standards of beauty and ugliness, good and evil, and cleanliness and pollution. There are also corresponding eating activities and specific food in specific festivals, emphasizing the promotion of festival atmosphere and so on, for example, turkey for Christmas in America, and dumplings for the Spring Festival in China.

Nowadays, dietary culture has developed by leaps and bounds. Modern science and technology are used to improve, cultivate, and produce new varieties of cooking raw materials, and promote and create new cooking tools. A new system has formed for modern dietary culture. People are pursuing both color and taste while paying attention to nutrition. However, some unhealthy eating behavior effected by dietary culture and beliefs still exists, such as eating raw/underprepared food or meat and products from wild animals, which could bring risks to human health.

## Eating behavior and infectious diseases

Reasonable eating behavior can provide adequate and balanced nutrition to our body. However, unhealthy eating behavior is closely related to the occurrence of diseases, not only chronic non-communicable diseases, but also infectious diseases. A number of studies have shown that unhealthy eating behavior has a certain impact on the occurrence and development of infectious disease as well as prevention and control, which is noteworthy. For example, consumption of rare or underprepared food is an important cause of infectious diseases.

With the improvement of living conditions, more people no longer seek to eat full, but to eat healthy. For example, it has become increasingly popular to eat raw food, marinated food, and drunk food, including meat products, aquatic products, and plant-based products, seafood in particular, which make a great contribution to foodborne infectious diseases.

To maintain the food’s quality, especially its delicious taste, people in some places like to eat raw food. They believe that fresh food has the richest nutrition and the best taste, and the best consumption period of any food is when its vitality is at its peak. Consumption of raw and improperly cooked food is very common in South and Southeast Asia [15]. For example, in Japan, where seafood resources are abundant and raw food culture is popular, more than 70% of foodborne illnesses have been linked to seafood [16], and about 1000 cases of anisakiasis are reported every year. In addition, some traditional cuisine believes that food processing technology should be minimized, and food should be eaten as close to the nature state as possible so that food could maintain its highest nutritional value. Nevertheless, there are huge health risks associated with raw or underprepared food, such as bacteria, viruses, parasites, or toxin and allergen. A number of varieties of parasites, including nematodes, trematodes, cestodes, cestodes, and protozoa [17], and viruses and bacteria, like human caliciviruses, hepatitis A virus, vibrio species, and *Streptococcus suis*, have been reported to cause infection outbreaks related to consumption of raw food, which can be seen all around the world. A case study in the lower Northern Thailand [18] found that daily consumption of raw fish averaged a 3.12–3.60 times higher risk of a liver fluke *Opisthorchis viverrini* infection compared to those with no raw fish eating habit. A report of hepatitis E infection in Jordan [19] showed that eating underprepared meat was significantly associated with HEV seropositivity (OR 2.06; 95% CI 1.04, 4.06). Park et al. [20] concluded that the habit of eating raw fish was the major factor for the maintenance of clonorchiasis among residents of riverside areas in Muju-gun, Jeollabuk-do, Korea; the odds ratio of residents who had eaten raw fish was 3.2-fold higher than that of those who had not.

**Fig. 1:**
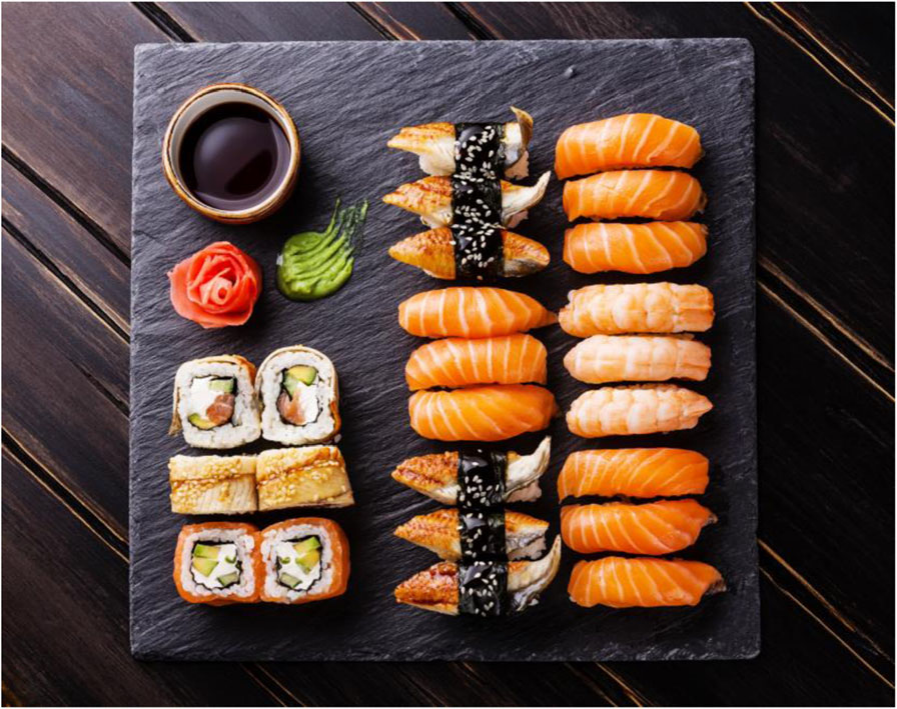
“Sushi”. Sushi is a traditional Japanese dish, usually made by cutting fresh sea urchin yellow, abalone, peony shrimp, scallops, salmon roe, cod roe, tuna, salmon, and other seafood into slices and placing them on the rice ball

**Fig. 2:**
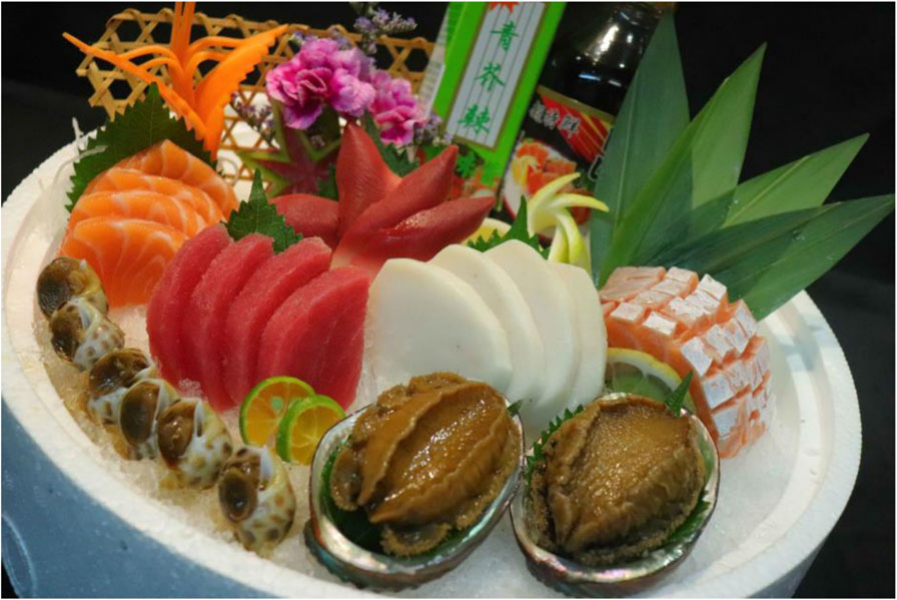
“Sashimi”. Sashimi is a fish dish in which fresh fish and shellfish are sliced and eaten directly with a sauce

Fish-borne zoonotic nematodes could infect human beings when fish or other aquatic products are consumed live, raw, smoked, or lightly cooked. In South America, potentially zoonotic nematode species are very common; infection with all kinds of nematodes was reported. Most of these cases were from countries such as Peru, Chile, and Ecuador where people have a tradition of eating raw or inadequately cooked aquatic products as in the form of the traditional dish called “ceviche.” Another important form was the raw fish prepared according to traditional Japanese dishes, like “sushi” (Fig. 1) and “sashimi” (Fig. 2) [21].

Clonorchiasis, also known as liver fluke disease, is endemic in China, Korea, Vietnam, and Japan. People at any age can be infected. It was one of the most serious foodborne parasitic diseases in China at the beginning of the twenty-first century. It was estimated that 35 million people worldwide were infected, of which about 15 million people in China [22]. Totally, 62 counties and cities have reported an epidemic of clonorchiasis in Guangdong province, China, and the most severe epidemic areas were mainly distributed in the Pearl River Delta region, especially Jiangmen [23]. The infection rate was 21.77% in Jiangmen, and it could reach 78.8% in some rural areas in 2007 [24]. Eating sashimi or raw or lightly cooked shrimp was the main risk factor related to clonorchis infection. Infection rate was higher in males, the reasons for which were indistinct but may be related to more opportunities of outside eating for males. Moreover, sashimi was often used as a treat to entertain relatives and friends, which further expanded the infection. Likewise, liver fluke disease is highly prevalent in rural southern Laos where raw food consumption had a deep cultural root. Their misunderstandings about health risks of raw food consumption were widespread. Raw fish with a “sour juice” made from squeezed adult weaver ants and spices like salt was considered to be equal to cooked fish. It was a long-standing tradition to use raw fish in various dishes, and the Laotians argued that eating these raw fish dishes means to keep the bonds of friendship and kinship [25]. Also, local people claimed that these dishes can supply them with strength and energy which were prerequisite for hard physical work. In addition, these dishes were usually prepared and served at many important social and cultural festivities or religious ceremonies where it was socially unacceptable to refuse to eat them [26].

Hepatitis A virus (HAV) is a single-stranded RNA virus surrounded by a protein capsid. The outbreaks of hepatitis A virus infection has happened worldwide [27], like Sweden, the USA, Australia, Italy, and so on. The infection was transmitted through contaminated water and foods via the fecal-oral route [28], and the largest raw seafood-related epidemic was reported from Shanghai, China. During the first quarter of 1988, an unprecedented major epidemic of hepatitis A occurred in Shanghai, China. Over 300,000 cases were reported, of which 47 (0.015%) were fatal [29]. This outbreak was traced to consumption of raw clams which was contaminated by domestic sewage and excrement [30]. A species of clams called *Anadara subcrenata lischke* was considered as the source of infection, which was supplied and transported from Qidong County, Jiangsu Province, where high incidence of hepatitis A was reported [31]. It was believed that the clams with some blood were more delicious. Eaten immediately after it was dipped in hot water, Shanghai’s blood clams reportedly had a raw, briny taste that stands out in a dietary culture that was all about freshness and mouth feel. However, this simple cooking was obviously not enough to kill the germs, so the bacteria and hepatitis A virus adsorbed on the gills of the clams invaded their digestive tract and liver easily.

**Fig. 3:**
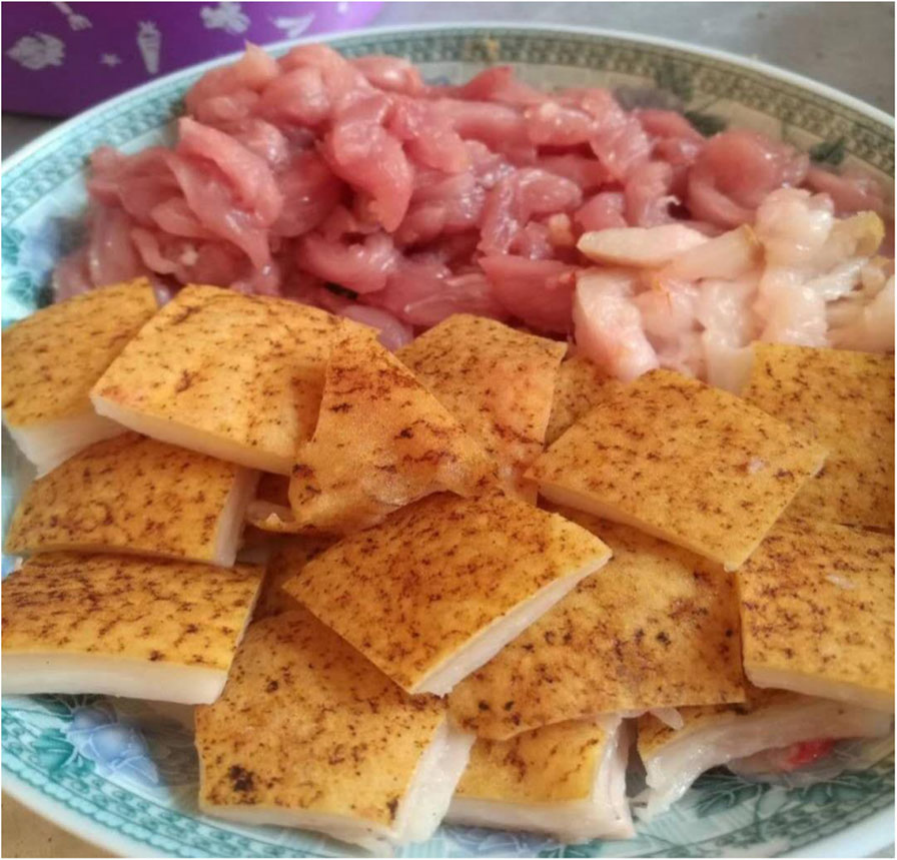
“Shengpi”(生皮). Shengpi from Dali, Yunnan Province, China. Shengpi is a traditional dish of Bai nationality. The Bai people always take it as their signature dish and specialty dish during the festival or the daily gathering. There are two ways to eat Shengpi, one is that meat and seasoning are not put together, and the other one is to mix seasoning and meat directly

**Fig. 4:**
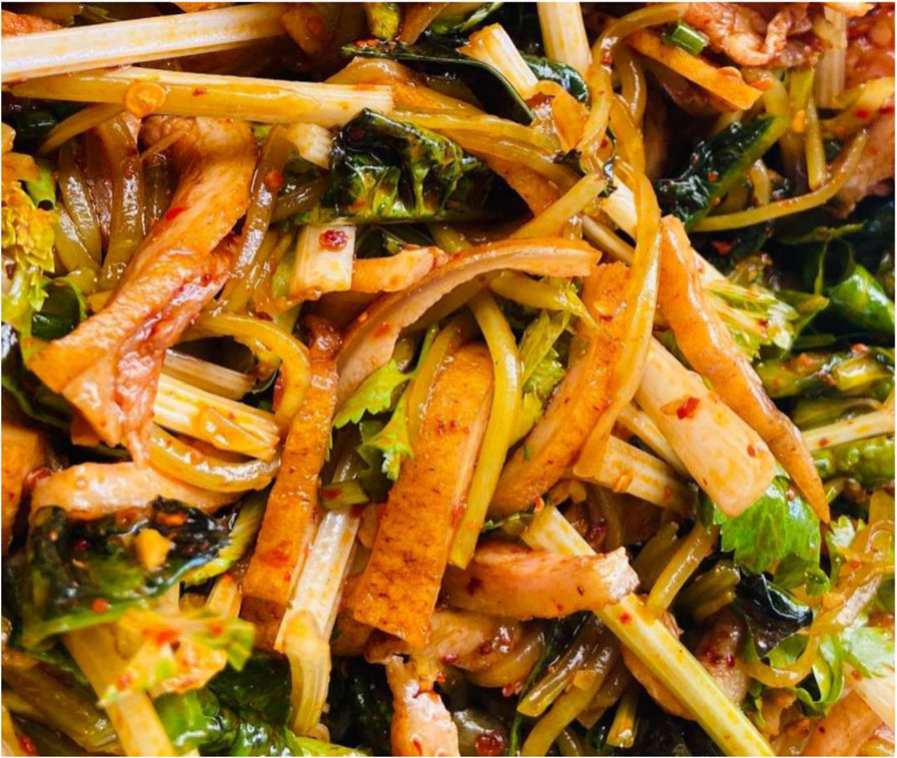
“Oru”(噢嚅). Oru from Hani nationality of Yunnan Province, China. It is cold vegetables with raw pork

**Fig. 5:**
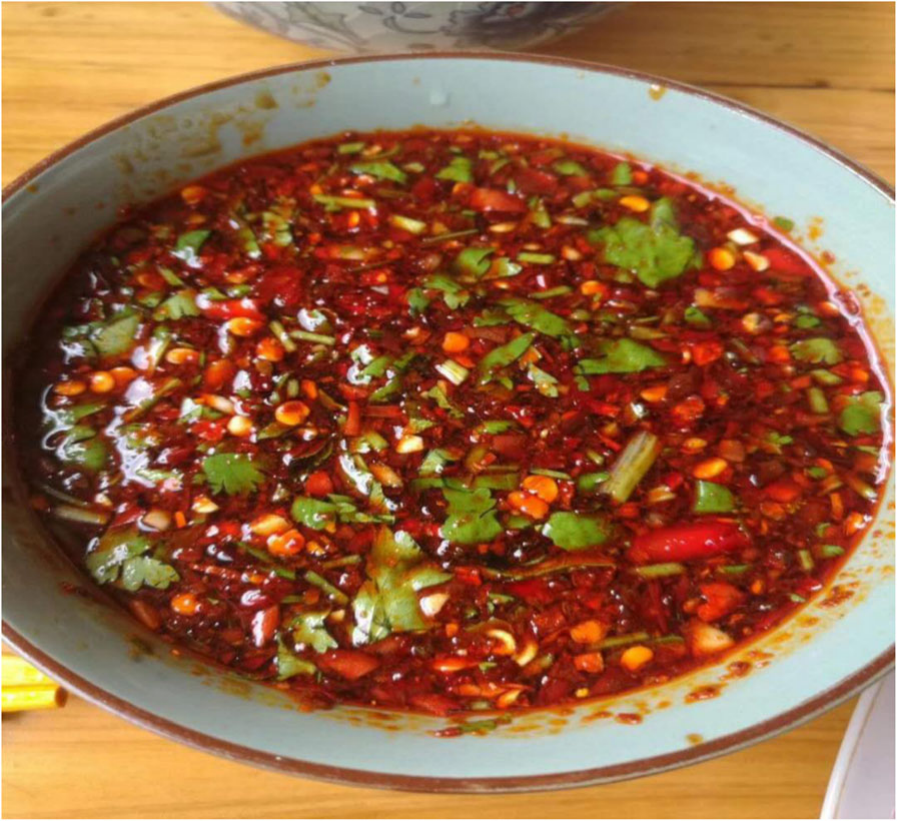
Seasoning. Seasoning is the soul of Yunan cuisine. The ingredients in it are not fixed but can be added or removed according to one’s taste. In general, seasoning includes pepper, star anise powder, chili powder, sesame powder, coriander, chives, and ginger

Cysticercus is the larva of the tapeworm *Taenia solium* and can cause cysticercosis in humans through intake of raw or underprepared pork contaminated with mature cysts [32]. Cysticercosis has been classified as one of the most important global foodborne parasites. Yunnan province is a serious cysticercosis epidemic area in China [33]. Local people have a habit of eating raw or undercooked pork or liver, which plays a decisive role in the spread of the disease. Some festival dishes in the minority areas of Yunnan province are made of raw pork, such as the “shengpi”(生皮) (Fig. 3) of Bai nationality, the “duosheng”(剁生) of Dai nationality, and the “oru”(噢嚅) (Fig. 4) of Hani nationality. The Bai people do not use boiling water but fire to kill pigs; they put the whole pig with fur on the fire until the fur is charred and scraped off, then cut the meat into thin slices and eat with seasoning (Fig. 5), which is called “Shengpi”(生皮). “Duosheng”(剁生) is a raw meat paste with seasoning. “Oru”(噢嚅) is cold vegetables with raw pork. In 2003, 4533 cysticercosis cases were found in Yunnan from hospitals which were at or above the county level, of which the Bai people accounted for 57.73% cases [34]. The previous studies found that 80.29% of the 1086 cysticercosis cases had a history of eating uncooked meat products [35], and 93.10% of the 1696 hospitalized cases of cysticercosis had a history of eating “shengpi”(生皮) [36]. According to survey, nearly 80% of the fresh pork purchased was used to make “shengpi”(生皮) in their daily life in a village of Dali, Yunnan province [37].

*Streptococcus suis* is a gram-positive strain of pathogenic bacteria and a pathogenic zoonotic factor [38]. In Vietnam, *Streptococcus suis* infection was one of the most common cause of acute bacterial meningitis in adults [39]. There was a dish called “tiet canh” (raw blood pudding) which was acclaimed wildly in Vietnam [3]. The major ingredients of “tiet canh” were raw pig blood and other animals’ blood mixed with minced pork tissues. *Streptococcus suis* was a common bacteria in “tiet canh” [40]. A case-control study conducted between May 2006 and June 2009 showed that risk factors identified for *Streptococcus suis* infection included eating “high risk” dishes such as undercooked pig blood (OR 2.22; 95% CI 1.15, 4.28) and pig intestine (OR 4.44; 95% CI 2.15, 9.15) [41]. In 2015, five swine streptococcus infections cases were reported, three of which were caused by eating raw blood pudding.

It is shocking to know that 75% of emerging infectious diseases originated from animals and no less than two-thirds of these originated in wildlife [42]. In modern times, though meat from wild animals is not needed for survival and it should not be part of dietary culture and modern civilization, the tradition of eating it persists in some places. The obsession with rare meat/animal products may stem from the philosophy of medicine food homology and the idea of when a thing is rare, it becomes precious. Many people mistakenly think that meat and products from wild animals not only taste good but also have nourishing effects. For example, penis of a deer is believed to have aphrodisiac effect, and the brain of some wild animals is supposed to make people smarter. Another false belief often held is that meat and products from wild animals have certain therapeutic effects. For instance, pangolin meat is believed to help relieve rheumatism; the bile of snakes is believed to improve eyesight. The spread of such misconceptions has made many people fall into a superstitious and inextricable zone. Meanwhile, rare and expensive food can be used by people to express wealth and social status [43]. The consumption of these foods seems to be more “characteristic” and “upper-grade,” so consumers have a display of economic capacity and social status, and their vanity is greatly satisfied.

Due to exposure to the wild and lack of quarantine, wild animals often carry a variety of bacteria and viruses and other pathogens. During the process of capturing and eating, these pathogens can be taken to human beings. More than 1400 kinds of pathogens are known to be able to infect humans. Among them, nearly 61% of the pathogens come from wild animals [44]. For example, bats can carry more than 130 highly pathogenic viruses [45], like lyssaviruses, Hendra virus, Nipah virus, coronaviruses, Ebola, and Marburg viruses. Moreover, humans in Africa are still exposed to a possibility of being infected by disease-causing microbes from wildlife via hunting and handling of bushmeat [46]. Bushmeat is a general term for “game” from the tropics, mainly referring to West and Central Africa. The meat is not from poultry or livestock, but directly from wild animals such as primates, rodents, or fruit bats found in tropical jungles. Tropical forests are known for their abundance of species, but these animals carry a great number of pathogenic microorganisms. Acquired immune deficiency syndrome (AIDS), for example, can be traced to chimpanzees [47].

Trichinellosis is a worldwide foodborne parasitic zoonotic disease caused by nematodes of the genus Trichinella, which are widespread globally in wildlife. Human infections are strictly related to cultures, where dietary habits include consumption of meat from wild animals. Many cases of trichinellosis have been reported in the Arctic (North Quebec, Nunavut, and Greenland) due to eating meat and products from black, brown, or polar bears [48]. In November 2014, a large outbreak of trichinellosis occurred in Belgium, related to the consumption of imported wild boar meat [49]. In 2011, an outbreak of trichinella infections associated with wild boar hunted at a game farm was reported in Iowa [50]. Growing interest in consuming wild boar has the potential to expose more individuals to this disease. In many Asian countries, many people were fond of eating meat and viscera of raw wild animals. This eating habit was one of the important factors in the transmission of taeniasis. Infections have been reported in Taiwan Province of China, Cheju Island of Korea, and Ambarita District on Samosir Island from 1984 to 1989. Among infected Taiwan aborigines, 73% had eaten wild boar, 66% flying squirrel, 65% wild goat, 56% muntjac, 49% wild rats, 46% monkey, 38% hare, 20% civet cats, 18% weasel, 17% pheasant, 14% squirrel, 4% grouse, 1% deer, 1% snake, less than 1% bamboo partridge, less than 1% frog, less than 1% bear, less than 1% dog, and less than 1% fox [51]. Furthermore, a case of *Escherichia coli* O157:H7 infection acquired by eating wild white-tailed deer was found in Connecticut [52]. And evidence suggested that eating wild boars is associated with a high risk of acquiring hepatitis E infection [53].

## Summary

Unhealthy eating behavior is related to the incidence and spread of some infectious diseases. Therefore, changing unhealthy eating behavior and practicing healthy eating behavior are very important in preventing and controlling the spread of infectious diseases. Some behaviors and habits may have been passed on from generation to generation, influenced by culture and beliefs. Obviously, it is difficult to change them completely within a short time, we need to make effort from multiple aspects, and special attention needs to be paid to the role of culture and beliefs. The ultimate solution lies in changing people’s conceptions about what is delicious, trendy, prestigious, or healthy to eat. Consequently, it is vital to popularize and spread healthy and scientific knowledge about diet to guide people to form the correct concept and develop healthy and scientific eating habits and lifestyles. Likewise, it is necessary to strengthen the cultivation and establishment of scientific and healthy food consumption concepts in early childhood. We can embed scientific diet into diet-related knowledge systems such as food education curriculum to help children establish a scientific and healthy food consumption concept as soon as possible.

## Data Availability

Not applicable.
